# Some Cases of Fever

**Published:** 1826-11

**Authors:** 

**Affiliations:** Physician to St. George's Hospital.


### FEVER.

### *Some Cases of Fever, treated upon the Principles lately explained** by Dr. Hewett, Physician to St. George's Hospital.

Case I.—William Noble, aged fifteen, was admitted into St. George's Hospital, on Saturday, the 15th August, 1826. The only information, left by his uncle, was that he had been ill with fever for five days, and delirious for the last three days.

Present symptoms are—a great prostration of strength; flushed face, but the pupils of the eyes are contractile; soft and feeble pulse, beating 120; dry, yellow, furred tongue; abdomen rather tense, and very tender in both iliac regions, but no complaint is made of pain there, except when pressure is forcibly applied; the skin is warm, but not pungently hot.

Applicentur Hirud. xx. abdomini quamprimum; postea descendat in thermas.—Hydrargyri Submuriatis, Pulveris Jacob. veri, āā gr. v.; Mucilag. Acaciæ q. s. ut fiant pilulæ duæ statim sumendæ; post tres horas sumatur Olei Ricini ℥ss. et repetatur quartâ quâque horâ, donec plenè dejecerit alvus.—Hydrargyri cum Cretâ ℈ss.; Pulveris radicis Ipecac. gr. j. M. fiat pulvis post catharsin sumendus, et repetendus ter die.—Radatur Capillitium et applicetur Lotio Spirituosa capiti.—Diæta febrilis.

13th.—Became very faint after the loss of blood by the leeches. Passed four evacuations unconsciously, and two this morning into the bed-pan. Was very noisy during the night. The face is still flushed; pulse is 120, very small, soft, and weak; skin of the body is warm, but that of the arms is cool; complains of great pain in his bowels when pressed upon, but not at other times; tongue is rough, brown, and dry almost to its edges, which are white and clammy.

Applic. Hirud. xij. abdom. deinde descendat in thermas; postea applicetur Emplastrum Lyttæ abdom.—Repet^r. Pulvis ter die.

14th.—Is less delirious. Has passed three or four well-coloured evacuations, and none of them involuntarily; but the abdomen is still exquisitely tender; pulse is 132, very small, soft, and weak; tongue is rough and dry, and he has great difficulty in protruding it; skin is moderately warm; and there is some slight cough.

Repet^r Pulvis ter die.—Haustus Salinus, Vin. Ipecac. m. xij. M. fiat haust. ter die sumendus.

—The uncle has this morning stated that his nephew walked with him to Islington on Sunday the 6th of August, but during the afternoon complained of great headache, and tottered, on his walk home, like a drunken man. His bowels were much relaxed, but not painful. He became delirious on Thursday the 10th of August, and remained so when admitted into the hospital.

15th.—Sulphat. Magnesiæ ℨiij. ex Haust. Salino statim.—Repet^r medicam.

16th.—Five well-coloured evacuations. Tongue slightly moist; abdomen still very tender; pulse 120, small, soft, and weak.

Repet^r medicam.

* See our Number for September.

17th.—Delirium has entirely disappeared. Bowels are freely open; pulse 112, of better size and strength; says he has no pain whatever, except when pressed upon the body, and then only from the rawness of the surface occasioned by the blister; skin is moderately warm and moist.

Repet[r] Pulvis, Hydrarg. et Ipecac. horâ somni tantùm.—Repet[r] Haustus.

18th.—Has no pain whatever, excepting that of blister. Tongue is moist and clean; appetite has returned this morning; gums are not the least affected.

Cataplasma lini abdom[i] applicand.—Omit[r] Pulvis et Haustus.—Infusi Rosæ ℥x.; Acid. Sulph. dil. m. vj.; Syrupi Aurantii, Tr. Humuli, āā ʒj. M. fiat haust. quater die sumendus.—Pulv. Ipecac. comp. gr. v. horâ somni.

23d.—Pulse is about ninety, and every thing going on favourably.

Sulphatis Quinæ gr. ij.; Extr. Gentianæ q. s. ut fiat pil. quartâ quâque horâ sumenda suprabibendo haustum nuper præscriptum.

Went out cured September 5th.

Case II.—Sophia Martin, aged sixteen, unmarried, was admitted ill with fever on the afternoon of Friday, August 11th, 1826.

A purgative dose of Calomel and Jalap was given immediately; and at night a pill, containing three grains of Calomel and the same quantity of James's Powder.—An evaporating lotion was applied to the head.

Saturday, August 12th.—Present symptoms are—flushed face; warm, but not dry skin; great pain on pressure of the abdomen, but at no other times; suffused eyes, but contractile pupils; tinnitus aurium; slight head-ache; no increased action of the temporal arteries; pulse 120, very soft; tongue and lips loaded with dark-brown fur; great deafness. She is quite incapable of giving the previous history of her symptoms. Her friends merely left word, that three weeks ago she caught cold, from remaining in wet clothes, during the catamenial period, by which the menstrual discharge was suddenly stopped; and that she has been very ill ever since.

Applicentur Hirudines xxx. abdom[i] statim; postea descendat in thermas.—Hydrargyri cum Cretâ ϑss.; Pulveris rad. Ipecac. gr. j. M. fiat pulvis ter die sumendus.—Haustus effervescens cum Tartrat. Sodæ ʒij. quotidie circa meridiem sumendus.

13th.—Was sleepless, but not noisy, during the night. The head, though still giddy when moved, is less so than yesterday. Bowels have acted freely; abdomen still very tender on pressure, but not painful at other times; pulse 120, soft; the black fur is breaking off from the lips and tongue.

Hirudines xv. abdom[i] statim; postea thermæ.—Repet. medicamenta.

14th.—She slept tolerably well, but was occasionally noisy during the night. Abdomen is no longer tender. One well-coloured evacuation; pulse 112, soft; skin moist and moderately warm; tongue still furred, but improving.

Repet. medicamenta.

17th.—Bowels have been freely open, but the abdomen, though not tender, still feels tumid. Pulse 116, very soft. Mouth is beginning to be affected by mercury.

Repet. Pulvis horâ somni tantùm.—Olei Ricini ℥ iij. cras primo mane.

18th.—Tongue is quite clean and moist; mouth is only very slightly affected. Several evacuations of well-coloured feculent matter, intermixed with white flakes; pulse 116, soft. She is still wakeful, but is obviously improving.

Pulv. Ipecac. Comp. ℈ss. o. n.—Infus. Rosæ ℥ x.; Acid. Sulph. dil. m. viij.; Syrupi Aurantii, Tr. Humuli, āā ʒj. M. fiat haust. quater die sumendus. —Omitt^r alia medicamenta.

19th.—Appetite has returned, but she is still wakeful at night. Pulse 108, small and feeble. The sister now informs me that, within eight or ten days of her admission, she had been twice bled; that her illness commenced, at the time already stated, with rigors, head-ache, flushed face, delirium, severe pains in the bowels, and diarrhœa.

20th.—Sulphate of Quina was given in addition to the above draught, under which treatment she gradually recovered, experiencing, however, occasional returns of pain and tenderness of the abdomen.

Case III.—Rebecca Stratton, aged seventeen, single, was admitted July 20th, 1825; but could give no further account of herself than that she was attacked, four weeks ago, with a cold fit, followed by headache. She delivers her answers very slowly. Tongue is moist, and skin natural. State of bowels is unknown, but abdomen feels full and loaded.

She was purged with Calomel and Rhubarb.

21st.—Passed a noisy night, crying out, she said, from pain in the head and body. The pupils of the eyes are much dilated, and scarcely contract on the approach of a candle close to them; face and lips pallid; tongue nearly clean; pulse very frequent and feeble; moans on pressure of the abdomen.

Applicetur Emplastrum Lyttæ nuchæ.—Hydrar. cum Cretâ gr. v. ter die.

22d.—Slept quietly; but pupils are still dilated and torpid, conjunctiva slightly vascular, and temporal arteries acting with some strength. The pulse is 120, soft and small; the evacuations are liquid, and free from blood; skin is moderately warm; moans on pressure of abdomen.

Applic. Hirudines xij. fronti, postea Lotio Spirituosa.—Hydrargyri cum Cretâ ℈ss. ter die.

23d.—Still shrinks on pressure of the abdomen, but slept quietly.—A female friend came to-day from Battersea to see her, and stated that, on the 11th July, Rebecca Stratton was brought to her house in a coach; that she was suffering from violent headache and fever; that a medical gentleman gave her some opening medicines, applied a blister to the nape of the neck, and, after a week, allowed her a gill of port-wine daily. She used to complain of much pain in her bowels, had frequent evacuations, and occasionally passed blood in them.

Repet. medicamenta.

27th.—Pupils slowly contractile, but the light is still painful to her eyes. Bowels have been kept freely open. She still winces on pressure of the abdomen. Pulse 100, soft and small.

Hydrargyri cum Cretâ ℈ss. quater die.

28th.—Pupils becoming still more contractile. Tongue is clean, but abdomen is still tender.

Repet$^{r}$ Hydrargyrum ter die.

August 1st.—No remains of head-ache; has recovered her appetite; but still the right eye cannot bear a strong light.

Repet$^{r}$ Hydrargyrum o. n.

3d.—Gums are slightly tumid and tender; pulse is small and feeble; but she is going on well in all respects.

Omitt$^{r}$ Hydrargyrum cum Cretâ.—Sulphatis Quinæ gr. ij.—Ext. Anthemidis q. s. ut fiat pil. quartâ quâque horâ sumenda.

8th.—Was allowed a small quantity of meat and wine; and went out cured August 14th.

Case IV.—Esther Stokes, aged seventeen, unmarried, was suddenly seized, May the 12th, with the usual symptoms of fever; on Sunday the 14th, took to her bed, with violent headache and hot skin; and, in four days more, became delirious and very noisy. No further information was left at the hospital by her friends respecting her.

May 24th —She is at present almost in a comatose state, but moans if forcible pressure is applied to the abdomen, which is tense. Urine and fæces have been passed involuntarily; the pupils of the eyes are dilated, but the face is not flushed, nor is there any increased action of the temporal vessels. Tongue is moist and clean; pulse is soft, small, and frequent; skin moderately warm.

Radatur Capillitium statim, et applic$^{r}$ Hirudines xvj. frontis; postea applic$^{r}$ Glacies capiti.—Hydrarg. Submur. gr. iv.; Scammoneæ gr. xij.; Sacchari albi q. s ut fiat pulvis statim sumendus, et repetendus post sex horas.

25th.—Remained quiet during the night. Several liquid and green evacuations. Abdomen is still tumid and tender. Pulse 116, soft and small; skin quite cool.

Hydrargyri cum Cretâ ℈ss. ter die.

26th.—Passed a noisy night, and is still delirious. The evacuations are passed unconsciously; abdomen is tympanitic and tender; pupils of the eyes are sluggish, but contractile; skin cool; pulse very frequent, small and feeble.

Repet$^{r}$ Hydrarg. cum Cretâ ℈ss. o. n. et m.

29th.—Has been noisy during the night. Face is flushed; pupils are again dilated. Four well-coloured and scarcely liquid evacuations. Abdomen still tympanitic and tender; pulse 144, soft and weak.

Hydrargyri Submur., Pulveris Jacobi veri, āā gr. ij.; Ext. Hyoscyami gr. iv. M. fiant pil. duæ sextis horis sumendæ.—Haust. Aquæ Ammoniæ Acetatis quater die.—Omittantur alia.

June 1st.—Six evacuations in the last twenty-four hours; none of them passed unconsciously. Abdomen is becoming supple, and no longer tender; pulse 116, small and soft; warm universal perspiration, and very obvious general amendment.

Repet[r] Pil. 8vâ quâque horâ.—Repet[r] Haustus.

3d.—Has had scarcely any sleep, but was not the least delirious. Abdomen is still puffy, but not the least tender; gums are scarcely affected.

Omitt[r] Pil.—Repet[r] Haustus.

5th.—Was rather noisy again during the night. Has had eight or nine liquid, but well-coloured evacuations. Tongue is quite clean and moist; pulse 116, soft and small.

Sulphatis Quinæ gr. ij. in formâ pilulæ quartâ quâque horâ sumendæ, superbibendo Haustum seq.—Acid. Sulph. dil. m. iv.; Syrupi Aurantii ʒ ij.; Tr. Cinnamomi ʒ ss.; Aquæ Pimentæ ʒ xj. M. Adde Tr. Opii m. v. haust. nocturno, si plus æquo soluta fuerit alvus.

6th.—Slept well. Had three evacuations before, but none since the addition of the Tr. Opii.

Was afterwards allowed three ounces of port wine daily; left her bed on the 15th, and the hospital on the 19th of June, cured.

In the two first of the preceding cases, it was imagined that the disturbance of the cerebral functions was kept up by sympathy with the diseased condition of the intestines, rather than from any source of irritation, such as vascular fulness or slight effusion of serum, in the brain. The remedies, therefore, were directed principally to the relief of the bowels.

The degree to which the mucous membrane of the intestines was affected, seemed to be less advanced in the first than in the second case. The invasion of the fever was so recent in the first, that the injury inflicted upon the mucous membrane could hardly be supposed to have exceeded that of enlargement of the mucous follicles, combined with partial inflammation of the neighbouring exhalant surface. This supposed existence of inflammatory action in the mucous membrane, seemed to indicate that the period for active and repeated purging was passed, and led to the adoption of the line of treatment, the principles of which have been already explained.

In the second case, the period of the fever, and the combination of symptoms existing at the time of her admission, impressed me with the conviction (afterwards still more fully confirmed by the history of the symptoms subsequently obtained, and by the occasional returns of abdominal tenderness and irritation during the slow progress to a state of convalescence,) that the structural lesion had advanced to the degree of the establishment of ulcerations in the mucous follicles, combined with partial inflammation of the contiguous exhalant surface of the intestines; the treatment pursued was in conformity with these views.

In the third case, the history of the previous symptoms, the dilatation of the pupils, the slowness and confusion of intellect, and the long-continued and severe headache, sufficiently show that the brain was directly suffering from pressure, either by effused fluid or vascular turgescence, in addition to the irritation transmitted to it by sympathy with the morbid (probably ulcerated) condition of the mucous membrane of the intestinal canal. The advanced stage of the fever, and the exhausted state of the patient, permitted the use only of mild measures: they were directed to the relief of the brain, as well as of the intestines; and it may be observed, that the mercurial treatment seems to have produced an equally beneficial effect upon both of these organs.

The fourth case affords an additional illustration of the same principles of treatment.

In all these cases it is sufficiently obvious that the period for the use of active purging was already past, at the time of their admission into the hospital. In the first case, this period had been unusually short, in consequence of the very early appearance of symptoms indicating some partial inflammations of the mucous membrane of the intestines.

It has been previously remarked, that the administration of powerful and repeated purgatives ought to be limited to the early stage of fever, and that the occurrence of symptoms indicating the existence either of ulcers or inflammation in the intestines demands their immediate discontinuance, and requires a corresponding variety in the treatment. But a bold and well-regulated succession of purgatives at the early period was strongly recommended, as affording the most effectual means of unloading the mucous glands, and thus anticipating the establishment either of ulcers or inflammation in the inner membrane of the bowels. To this system an objection, more specious than just, is sometimes urged,—viz. that this free use of purgatives frequently excites or accelerates the very evils, which it is intended to avert. The opponent undoubtedly has so far the advantage of position, that wherever, in fatal cases of fever, ulcers are discovered in the intestines *after* the purgative treatment above recommended, they may very plausibly be imputed to it; while its operation, where successful, necessarily destroys all opportunity of adducing any palpable anatomical evidence in its favour. The fallacy, however, of this objection is easily shown; for a very limited experience in the dissection of those, who die the victims of fever, is sufficient to detect the frequent existence of such ulcers in the intestines, when only the mildest plan of treatment had been previously pursued. Thus a young girl, Caroline Spooner, was admitted into St. George's Hospital, on the 3d of August, 1826, in such a perfectly hopeless state of fever, that none but soothing measures were adopted. She died on the 6th August. I was indebted to the kindness of the medical gentleman, who had attended her, for the particulars of the previous treatment: they consisted of diaphoretics, occasional mild doses of blue-pill, and chalk mixture. Upon examination after death, the *small* intestines were found most extensively affected with follicular ulcers.

Henry Long, aged twenty-five, was admitted under my care on the afternoon of Thursday, August 24th, 1826, having been suddenly attacked with fever about fourteen or sixteen days previously. He had suffered much from headache, for which he had been twice bled: the pain was removed, but a constant giddiness remained. His bowels had been kept freely open, but by no means violently purged. Neither at the time of his admission, nor until the Monday afternoon, (although he had been daily submitted to forcible pressure of the abdomen,) did he acknowledge any pain, or even uneasiness, of the bowels; but, at four o'clock P. M. of Monday the 28th, he was suddenly seized, while passing an evacuation, with a most excruciating pain and exquisite tenderness of the abdomen: leeches and fomentations appeared to produce some little alleviation of his sufferings. On the morning visit of Tuesday, it was stated, and immediately recorded in the hospital case-book, that a perforation of the peritoneum had been effected by the progress of a follicular ulcer from the mucous membrane of the intestines, and that peritonitis had thus been excited. The circulation was then scarcely perceptible in the extremities, and he expired at half after two o'clock P.M. of Tuesday, the 29th. The *sectio cadaveris* took place on the following day. Upon opening the cavity of the abdomen, very extensive peritonitis was observed, and a follicular ulcer was discovered within three inches of the ileo-cœcal valve, perforating all the coats of the ileum. In its neighbourhood there was another ulcer, just upon the point of effecting the perforation of the peritoneum; and along the whole course of the ileum there were several similar ulcers, exhibiting the progressive stages of ulceration. The specimen is preserved, and is ready to attest the accuracy of this statement.

It would be very easy, if it were not now both tedious and unnecessary, to accumulate examples of the frequent origin of these ulcers, during the progress of fever, in the mucous membrane of the *small* intestines, quite independently of any irritation produced by an abuse of purgatives.

*September*, 1826.

